# Ethical governance is essential to building trust in robotics and artificial intelligence systems

**DOI:** 10.1098/rsta.2018.0085

**Published:** 2018-10-15

**Authors:** Alan F. T. Winfield, Marina Jirotka

**Affiliations:** 1 Bristol Robotics Laboratory, University of the West of England, Coldharbour Lane, Bristol BS16 1QY, UK; 2 Department of Computer Science, University of Oxford, Parks Road, Oxford OX1 3QD, UK

**Keywords:** artificial intelligence ethics, robot ethics, governance, standards, responsible innovation, trust

## Abstract

This paper explores the question of ethical governance for robotics and artificial intelligence (AI) systems. We outline a roadmap—which links a number of elements, including ethics, standards, regulation, responsible research and innovation, and public engagement—as a framework to guide ethical governance in robotics and AI. We argue that ethical governance is essential to building public trust in robotics and AI, and conclude by proposing five pillars of good ethical governance.

This article is part of the theme issue ‘Governing artificial intelligence: ethical, legal, and technical opportunities and challenges’.

## 1. Introduction

The aim of this paper is to present a case for a more inclusive, transparent and agile form of governance for robotics and artificial intelligence (AI) in order to build and maintain public trust and to ensure that such systems are developed for the public benefit. Building public trust in intelligent autonomous systems (IAS) is essential. Without that trust, the economic and societal benefits of IAS will not be realized. In this paper, we will lay out a roadmap. The value of a roadmap is twofold; firstly, it connects and maps the different elements that each contribute to IAS ethics, and secondly it provides us with a framework to guide ethical governance.

Over the last decade, the private sector has made significant investments in the development of robots and AI with autonomous capacities that can interact with humans in order to fulfil roles in work, home, leisure, healthcare, social care and education. These developments potentially offer huge societal benefits. They can save time, reduce human effort to perform tasks and reduce costs. They can also improve well-being through the provision of reliable care assistance for the ageing population, standardization in service encounters, companionship and affective aids for different user groups, and relieve humans from both dangerous and menial tasks.

Public attitudes towards these new intelligent technologies are generally positive [1,2]. However, concerns have been raised regarding the irresponsible use and potentially harmful impact of IAS. These concerns are frequently raised in public rhetoric, which tends to position robots' future ubiquity as inevitable and construct dystopian scenarios featuring the usurpation of human autonomy, safety and authority. At the same time, there are genuine—and possibly well founded—fears around the impact on jobs and mass unemployment [3]. We know that there is no ‘formula’ for building trust, but we also know from experience that technology is, in general, trusted if it brings benefits and is safe and well regulated.

Building such trust in robotics and AI will require a multiplicity of approaches, from those at the level of individual systems and application domains [4] to those at an institutional level [5,6]. This paper argues that one key (necessary but not sufficient) element in building trust in IAS is ethical governance. We define ethical governance as a set of processes, procedures, cultures and values designed to ensure the highest standards of behaviour. Ethical governance thus goes beyond simply good (i.e. effective) governance, in that it inculcates ethical behaviours in both individual designers and the organizations in which they work. Normative ethical governance is seen as an important pillar of responsible research and innovation (RI), which ‘entails an approach, rather than a mechanism, so it seeks to deal with ethical issues as or before they arise in a principled manner rather than waiting until a problem surfaces and dealing with it in an ad hoc way’ [7].

Given the increasing pace of innovation [8], new and agile processes of governance are needed. A recent World Economic Forum (WEF) white paper has suggested that the rapid pace of transformative technological innovation is ‘reshaping industries, blurring geographical boundaries and challenging existing regulatory frameworks’ [9]. Increasingly, not only policy-makers, but also businesses and innovators, feel a duty to engage with policies to address the societal consequences of their innovation; the report calls for a more inclusive and agile form of governance. The WEF, as the international organization for public–private cooperation, is launching a global initiative on agile governance dedicated to reimagining policy-making for the fourth industrial revolution [10]. The WEF defines agile governance as ‘adaptive, human-centred, inclusive and sustainable policy-making, which acknowledges that policy development is no longer limited to governments but rather is an increasingly multi-stakeholder effort’ [11]. It is those non-governmental stakeholders, including individual researchers, research institutions and funders, professional bodies, industry and civil society, that are key to making ethical governance both agile and practical. In practice, this means incorporating different kinds of knowledge, including that from citizens, to inform the goals and trajectories of innovation.

This paper is concerned with the ethical governance of both physical robots, such as driverless cars or personal assistant robots (for care or in the workplace), and software AIs such as medical diagnosis AIs or personal digital assistants. All of these are intelligent agents with some degree of autonomy, so we refer to them collectively as ‘intelligent autonomous systems’ (IAS). The last 18 months have seen a proliferation of new ethical principles for robots and AI (especially AI). But principles are not practice and, while it is heartening to witness a growing awareness of the need for ethics, there is little evidence of good practice in ethical governance. Given that transparency is a core principle of ethical governance, one has to be sceptical of any claims that organizations make unless they, for instance, publish the terms of reference and membership of ethics boards, alongside evidence of good ethical practice. The gap between principles and practice is an important theme of this paper and the five pillars of good ethical governance that we propose in this paper are aimed at addressing this gap.

The paper is structured as follows. In §2, we build a roadmap to show how the components of ethical governance, including ethical principles, responsible innovation, standards and regulation, are connected. Then §3 provides a commentary on the roadmap, considering public fears, standards and regulation, safety-critical AI, transparency and moral machines. A brief concluding discussion—including a set of five recommendations for ethical governance—is given in §4.

## 2. Building the roadmap

**Figure 1.**
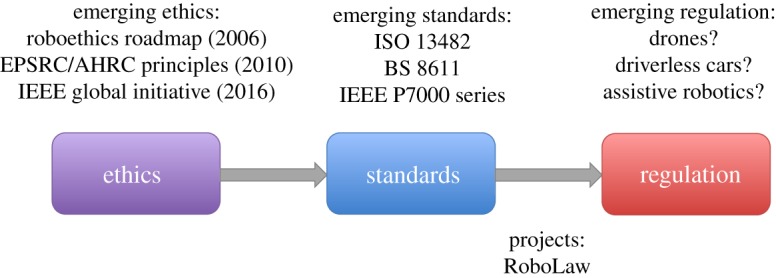
Linking ethics, standards and regulation.

The core of our roadmap connects research on ethics with emerging standards and regulation. Standards often formalize ethical principles into a structure which could be used either to evaluate the level of compliance or, more usefully perhaps for ethical standards, to provide guidelines for designers on how to reduce the likelihood of ethical harms arising from their product or service. Ethical principles may therefore underpin standards either explicitly or implicitly. Consider safety standards such as ISO 13482 [12]—here the underpinning ethical principle is that personal care robots must be safe. In ISO 13482 that principle is explicit but in many standards it is not. Process standards such as the ISO 9000 family of quality management standards could, for instance, be said to express the principle that shared best practice benefits all. But standards also sometimes need teeth, i.e. regulation which mandates that systems are certified as compliant with standards, or parts of standards. Most standards are voluntary. There is no requirement to adopt IEEE 802.11 (WiFi), for instance, in a new networked product, but not to do so would clearly be commercially unwise. And those standards that are mandated—often because they relate to safety—are *de facto* directed because a licence to operate a system would not be granted until after that system has been shown to be compliant with those standards. Furthermore, *soft governance* plays an important role in the adoption of standards: by requiring compliance with standards as a condition of awarding procurement contracts, governments can and do influence and direct the adoption of standards—across an entire supply chain—without explicit regulation. Accepting that this is a simplification of a process with many intervening factors, we argue that ethics (or ethical principles) lead to standards, which in turn lead to regulation, as shown in figure 1, and that this characterization has value in understanding the landscape of ethical governance.

Figure 1 references some foundational ethical frameworks, including the 2006 EURON Roboethics Roadmap [13] and the EPSRC Principles of Robotics [14]. An informal survey at the end of 2017 [15] discovered that a total of 10 different sets of ethical principles (including Asimov's Laws of Robotics^1^ ) had been proposed by December 2017, seven of which appeared in 2017. These are listed in table 1.

**Table 1.** Principles of robotics and AI published by December 2017.

principles	# principles	year and refs
Asimov's Laws of Robotics	3	1950
Murphy and Wood's three Laws of Responsible Robotics	3	2009 [16]
The EPSRC Principles of Robotics	5	2011 [14]
Future of Life Institute's Asilomar Principles for Beneficial AI	23	Jan 2017
ACM US Public Policy Council's Principles for Algorithmic Transparency and Accountability	7	Jan 2017
Japanese Society for Artificial Intelligence (JSAI) Ethical Guidelines	9	Feb 2017
The Future Society's Science, Law and Society Initiative draft principles	6	Oct 2017
Montreal Declaration for Responsible AI draft principles	7	Nov 2017
IEEE General Principles of Ethical Autonomous and Intelligent Systems	5	Dec 2017 [17]
UNI Global Union Top 10 Principles for Ethical AI	10	Dec 2017

There is a good deal of commonality across these principles, notably that IAS should (i) do no harm, including being free of bias and deception, (ii) respect human rights and freedoms, including dignity and privacy, while promoting well-being, and (iii) be transparent and dependable while ensuring that the locus of responsibility and accountability remains with their human designers or operators. Perhaps the most important observation relates not to the content of these principles but to the increasing frequency of their publication: clear evidence for a growing awareness of the urgent need for ethical principles for IAS. But principles are not practice. They are an important and necessary foundation for ethical governance, but only the first step.

In figure 1, we reference recent standards such as ISO 13482 [12] (Safety requirements for personal care robots) and—perhaps the world's first ethical standard for robotics—BS 8611:2016 [18]. Whereas ISO 13482 is concerned with personal care robots, the scope of BS 8611 extends to all classes and domains of robots and robotic systems.

Ethics and standards both fit within a wider overarching framework of responsible research and innovation (RI). RI initiatives across policy, academia and legislation emerged over a decade ago and began with an aim to identify and address uncertainties and risks associated with novel areas of science. This has recently expanded to consider computer science, robotics, informatics and information and communications technology (ICT) more generally. RI proposes a new process for research and innovation governance [19]. The aim is to ensure that science and innovation are undertaken in the public interest by incorporating methods for encouraging more democratic decision-making through greater inclusion of wider stakeholder communities that might be directly affected by the introduction of novel technologies.

**Figure 2.**
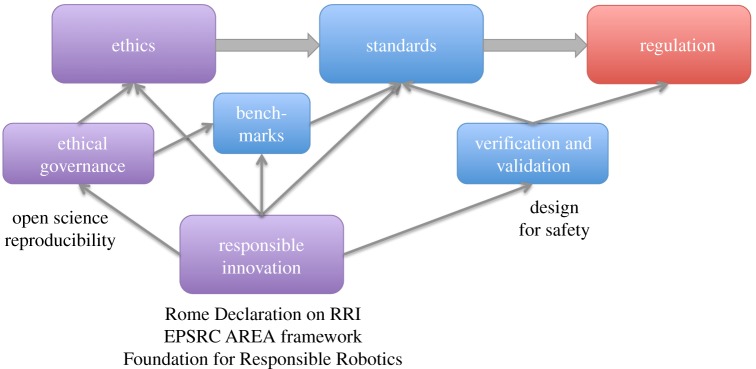
Scaffolded by responsible research and innovation.

Responsible innovation both informs and underpins ethics and standards, as shown in figure 2. Importantly, ethical governance is a key pillar of RI. RI also connects directly with ethics through, for instance, public engagement, open science and inclusivity; notably, open science has been described as a ‘trust technology’ [20]. Another key component of RI is the ability to systematically and transparently measure and compare system capabilities, typically with standardized tests or benchmarks [21].

A further key element of RI, especially when systems move into real-world application, is the need for verification and validation, to provide assurance both of safety and fitness for purpose. Verification and validation might be undertaken against published standards, and—for safety-critical systems—conformance with those standards may be a legal requirement without which the system would not be certified. Hence verification and validation link to both standards and regulation. Figure 2 references underpinning frameworks for responsible innovation including the 2014 Rome Declaration on Responsible Research and Innovation [22], the EPSRC Anticipate, Reflect, Engage and Act (AREA) framework [19,23] and the recently established Foundation for Responsible Robotics [24]. Furthermore, the AREA framework has been tailored specifically for ICT [25,26].

In general, technology is trusted if it brings benefits while also safe, well regulated and, when accidents happen, subject to robust investigation. One of the reasons we trust airliners, for example, is that we know that they are part of a highly regulated industry with an outstanding safety record. The reason commercial aircraft are so safe is not just good design, it is also the tough safety certification processes and, when things do go wrong, robust and publicly visible processes of air accident investigation. It is reasonable to suggest that some robot types, driverless cars for instance, should be regulated through a body similar to the Civil Aviation Authority (CAA), with a driverless car equivalent of the Air Accident Investigation Branch. It is important to note that air accident investigations are social processes of reconstruction that need to be perceived as impartial and robust, and which serve as a form of closure so that aviation does not acquire an enduring taint in the public consciousness. We anticipate very similar roles for investigations into robot accidents [27].

**Figure 3.**
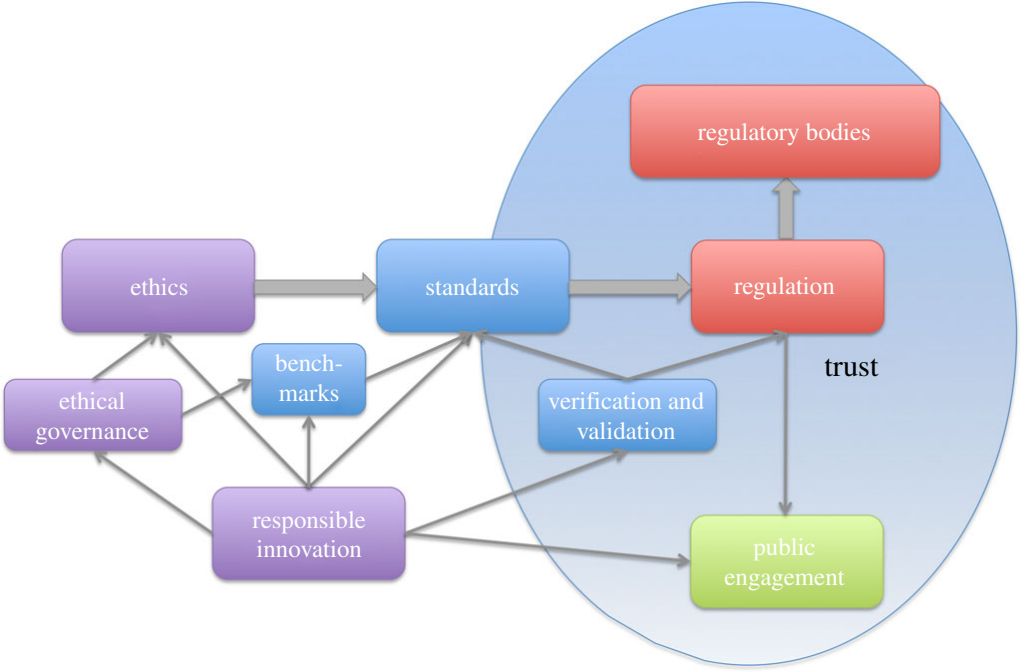
Building public trust.

Regulation requires regulatory bodies, linked with public engagement [28,29] to provide transparency and confidence in the robustness of regulatory processes. All of which supports the process of building public trust, as shown in figure 3.

## 3. Commentary on the IAS ethics roadmap

In the following sections, we provide a deeper commentary on a number of aspects of the roadmap touched on above, including public fears, standards and regulation. We also deepen and extend the present and future context of the roadmap with an introduction to safety-critical AI, the need for transparency, and—looking to the future—the governance issues of moral machines (systems that explictly reason about ethics).

### (a) Public fears

It is well understood that there are public fears around robotics and artificial intelligence. Many of these fears are undoubtedly misplaced, fuelled perhaps by press and media hype, but some are grounded in genuine worries over how the technology might impact, for instance, jobs or privacy.

The most recent Eurobarometer survey on autonomous systems showed that the proportion of respondents with an overall positive attitude has declined from 70% in the 2012 survey [1] to 64% in 2014 [2]. Notably, the 2014 survey showed that the more personal experience people have with robots, the more favourably they tend to think of them; 82% of respondents have a positive view of robots if they have experience with them, whereas only 60% of respondents have a positive view if they lack robot experience. Also important is that a significant majority (89%) believe that autonomous systems are a form of technology that requires careful management.

A recent survey of decision-making in driverless cars reveals distinctly ambivalent attitudes: ‘… participants approved of utilitarian Autonomous Vehicles (AVs) (that is, AVs that sacrifice their passengers for the greater good) and would like others to buy them, but they would themselves prefer to ride in AVs that protect their passengers at all costs. The study participants disapprove of enforcing utilitarian regulations for AVs and would be less willing to buy such an AV’ [30].

It is clear that public trust in IAS cannot simply be assumed [31–33]; to do so could risk the kind of public rejection of a new technology seen (in Europe) with genetically modified foods in the 1990s [34]. Proactive actions to build public trust are needed, including, for example, the creation of a ‘machine intelligence commission’ as argued by Mulgan [6]; such a commission would lead public debates, identify risks and make recommendations to Parliament, for new regulation or regulatory bodies, for instance, and recommend independent mechanisms for responsible disclosure.

### (b) Standards and regulation

Work by the British Standards Institution Technical Subcommittee on Robots and Robotic Devices led to publication—in April 2016—of BS 8611: *Guide to the ethical design and application of robots and robotic systems* [18]. BS 8611 incorporates the EPSRC Principles of Robotics [14]; it is not a code of practice, but instead gives ‘guidance on the identification of potential ethical harms and provides guidelines on safe design, protective measures and information for the design and application of robots’. BS 8611 articulates a broad range of ethical hazards and their mitigation, including societal, application, commercial/financial and environment risks, and provides designers with guidance on how to assess and then reduce the risks associated with these ethical hazards. The societal hazards include, for example, loss of trust, deception, privacy and confidentiality, addiction and unemployment.

The primary output from the IEEE Standards Association's global ethics initiative [35] is a discussion document called *Ethically aligned design* (EAD), now in its second iteration [17]. The work of 13 committees, EAD covers: general (ethical) principles; how to embed values into autonomous intelligent systems; methods to guide ethical design and design; safety and beneficence of artificial general intelligence and artificial superintelligence; personal data and individual access control; reframing autonomous weapons systems; economics and humanitarian issues; law; affective computing; classical ethics in AI; policy; mixed reality; and well-being. EAD articulates a set of over 100 ethical issues and recommendations. Each committee was asked to recommend issues that should be addressed through a new standard. At the time of writing, 14 standards working groups are drafting candidate standards to address an ethical concern articulated by one or more of the 13 committees outlined in EAD: the so-called IEEE P7000 ‘human’ standards. To give one example, IEEE P7001 *Transparency in autonomous systems* is drafting a set of measurable, testable levels of transparency for each of several stakeholder groups, including users, certification agencies and accident investigators [36].

Significant recent work on surveying the state of robotics regulation was undertaken by the EU project RoboLaw. The primary output of that project is a comprehensive report entitled *Guidelines on regulating robotics* [37]. That report reviews both ethical and legal aspects; the legal analysis covers rights, liability and insurance, privacy and legal capacity. The report focuses on driverless cars, surgical robots, robot prostheses and care robots, and concludes by stating: ‘The field of robotics is too broad, and the range of legislative domains affected by robotics too wide, to be able to say that robotics by and large can be accommodated within existing legal frameworks or rather require a *lex robotica*. For some types of applications and some regulatory domains, it might be useful to consider creating new, fine-grained rules that are specifically tailored to the robotics at issue, while for types of robotics, and for many regulatory fields, robotics can likely be regulated well by smart adaptation of existing laws.’

### (c) Safety-critical artificial intelligence

Initial efforts towards ethics and regulation was focused on robotics; it is only more recently that attention has turned towards AI ethics. Because robots are physical artefacts, they are undoubtedly more readily defined and hence regulated than distributed or cloud-based AIs. This and the already pervasive applications of AI (in search engines, machine translation systems or intelligent personal assistant AIs, for example) strongly suggest that greater urgency needs to be directed towards considering the societal and ethical impact of AI, including the governance and regulation of AI.

A reasonable definition of a modern robot is ‘an embodied AI’ [38]. Thus in considering the safety of robots, we must also concern ourselves with the AI controlling the robot. The three types of robot indicated in figure 1, drones, driverless cars and assistive robots,^2^ will all be controlled by an embedded AI,^3^ of some appropriate degree of sophistication. Yet these are all *safety-critical* systems, the safety of which is fundamentally dependent on those embedded AIs; decisions made by these embedded AIs have real consequences to human safety or well-being (in that a failure could cause serious harm or injury). Let us consider two general issues with AI, (i) trust and transparency and (ii) verification and validation, both of which come into sharp focus for our three exemplar robot categories of drones, driverless cars and assisted-living robots.

AI systems raise serious questions over trust and transparency:

— How can we trust the decisions made by an IAS and, more generally, how can the public have confidence in the use of AI systems in decision-making?
— If an IAS makes a decision that turns out to be disastrously wrong, how do we investigate the logic by which the decision was made, and who is responsible (noting that the AI cannot itself be responsible)?

Existing safety-critical systems are not AI systems, nor do they incorporate AI systems. The reason is that AI systems (and in particular machine learning systems) are largely regarded as impossible to verify for safety-critical applications. The reasons for this need to be understood.

— First is the problem of verification of systems that learn. Current verification approaches typically assume that the system being verified will never change its behaviour, but a system that learns does—by definition—change its behaviour, so any verification is likely to be rendered invalid after the system has learned.
— Second is the *black box* problem. Modern AI systems, and especially the ones receiving the greatest attention, so-called deep learning systems, are based on artificial neural networks (ANNs). A characteristic of ANNs is that, after the ANN has been trained with datasets,^4^ any attempt to examine the internal structure of the ANN in order to understand why and how the ANN makes a particular decision is more or less impossible. The decision-making process of an ANN is opaque.

The problem of verification and validation of systems that learn may not be intractable, but is the subject of current research; see, for example, work on verification and validation of autonomous systems [39]. The black box problem may be intractable for ANNs, but could be avoided by using algorithmic approaches to AI (i.e. that do not use ANNs). Notably, a recent report has recommended that ‘core public agencies … no longer use “black box” AI and algorithmic systems’ [40].

### (d) Transparency

One aspect of ethical governance discussed above is ‘transparency’. Transparency is an essential property of ethical governance; it would be hard to argue that opaque governance is ethical. Ethical governance in robotics and AI should ideally demonstrate both transparency of *process* and transparency of *product*; the former refers to the transparency of the human processes of research and innovation, the latter to the transparency of the robot or AI systems so developed.

Consider now product transparency. This will necessarily mean different things to different stakeholders—the kinds and levels of transparency required by a safety certification agency or an accident investigator will clearly need to be different from those required by the system's user or operator. Ideally, systems should be explainable, or even capable of explaining their own actions (to non-experts) as well as transparent (to experts). There is a growing literature on transparency; see, for instance, work on transparency and explainability in robot systems [27,41,42], transparency in relation to the EU General Data Protection Regulation (GDPR) [43,44] and on the limitations of transparency [45].

An important underlying principle is that it should always be possible to find out why an autonomous system made a particular decision (especially if that decision has caused or might cause harm). Given that real-world trials of driverless car autopilots have already resulted in several fatal accidents [46,47], there is clearly an urgent need for transparency in order to discover how and why those accidents occurred, remedy any technical or operational faults, and establish accountability. A new IEEE standard P7001 *Transparency in autonomous systems* is currently under development, which will ‘provide a guide for self-assessing transparency during development and suggest mechanisms for improving transparency’ [48].

A technology that would provide such transparency, especially to accident investigators, would be the equivalent of an aircraft flight data recorder (FDR). We call this an ethical black box (EBB) [27], both because aircraft FDRs are commonly referred to as black boxes,^5^ and because such a device would be an integral and essential physical component supporting the ethical governance of IAS. Like its aviation counterpart, the EBB would continuously record sensor and relevant internal status data so as to greatly facilitate (although not guarantee) the discovery of why a robot or AI made a particular decision or series of decisions—especially those leading up to an accident. EBBs would need to be designed—and certified—according to standard industry-wide specifications, although it is most likely that each application domain would have a different standard; one for driverless vehicles, another for drones and so on.

### (e) Towards moral machines

This paper is primarily concerned with robot and AI ethics, rather than ethical robots. But inevitably near-future autonomous systems, most notably driverless cars, are *by default* moral agents. It is clear that both driverless cars and assistive (i.e. care) robots make decisions with ethical consequences, even if those robots have not been designed to explicitly embed ethical values and moderate their choices according to those values. Arguably all autonomous systems implicitly reflect the values of their designers or, even more worryingly, training datasets (as dramatically shown in AI systems that demonstrate human biases [49]).

Moor [50] makes the useful distinction between *implicit* ethical agents, that is machines designed to avoid unethical outcomes, and *explicit* ethical agents, that is machines which either directly encode or learn ethics and determine actions based on those ethics. There is a growing consensus that near-future robots will, as a minimum, need to be designed to reflect the ethical and cultural norms of their users and societies [18,35], and an important consequence of ethical governance is that *all* robots and AIs should be designed as implicit ethical agents.

Beyond reflecting values in their design, a logical (but technically very challenging) next step is to provide intelligent systems with an *ethical governor*. That is, a process which allows a robot or AI to evaluate the consequences of its (or others') actions and modify its own actions according to a set of ethical rules.^6^ Developing practical ethical governors remains the subject of basic research and presents two high-level challenges: (i) the philosophical problem of the formalization of ethics in a format that lends itself to machine implementation and (ii) the engineering problem of the implementation of moral reasoning in autonomous systems [51–53].

There are two approaches to addressing the second of these challenges [54]:

(i) a constraint-based approach—explicitly constraining the actions of an AI system in accordance with moral norms; and
(ii) a training approach—training the AI system to recognize and correctly respond to morally challenging situations.

The training approach is developed for an assistive robot in [55], while examples of constraint-based approaches are explored in [56,57]. One advantage of the constraint-based approach is that it lends itself to verification [58].

Note that equipping future IAS with an ethical governor will not abrogate or diminish the human responsibility for careful ethical governance in the design and application of those systems. Rather the opposite: robots and AIs that are explicit moral agents are likely to require a greater level of operational oversight given the consequences of such systems making the wrong ethical choice [59]. Explicitly, ethical machines remain, at present, the subject of basic research; if and when they become a practical reality, there is no doubt that radical new approaches to regulating such systems will be needed.

## 4. Concluding discussion

In this paper, we have argued that, while there is no shortage of sound ethical principles in robotics and AI, there is little evidence that those principles have yet translated into practice, i.e. effective and transparent ethical governance. Ethical practice starts, of course, with the individual, and emerging professional codes of ethical conduct, such as the recently published ACM code [60], are very encouraging. But individuals need to be supported and empowered by strong institutional frameworks and principled leadership. What would we expect of robotics and AI companies or organizations who claim to practice ethical governance? As a starting point for discussion we propose five pillars of good ethical governance, as follows:

— Publish an *ethical code of conduct*, so that everyone in the organization understands what is expected of them. This should sit alongside a ‘whistleblower’ mechanism which allows employees to be able to raise ethical concerns (or ‘responsible disclosure’), if necessary in confidence via an ombudsperson, without fear of displeasing a manager.
— Provide *ethics and RI training* for everyone, without exception. Ethics and responsible innovation, like quality, is not something that can be implemented as an add-on; simply appointing an ethics manager, for instance, while not a bad idea, is not enough.
— Practice *responsible innovation*, including the engagement of wider stakeholders within a framework of anticipatory governance (using for instance the AREA framework [19,23,26]). Within that framework, undertake *ethical risk assessments* of all new products, and act upon the findings of those assessments. A toolkit, or method, for ethical risk assessment of robots and robotic systems exists in British Standard BS 8611 [18], and new process standards, such as IEEE P7000 *Model process for addressing ethical concerns during system design*, are in draft.
— Be *transparent* about ethical governance. Of course, robots and AIs must be transparent too, but here we mean transparency of process, not product. It is not enough for an organization to claim to be ethical; it must also show *how* it is ethical. This could mean an organization publishing its ethical code of conduct, membership of its ethics board if it has one (and its terms of reference), and ideally case studies showing how it has conducted ethical risk assessments alongside wider processes of anticipatory governance—these might be part of an annual *transparency report*.
— Really *value* ethical governance. Even if an organization has the four processes above in place, it—and especially its senior managers—also needs to be sincere about ethical governance; that ethical governance is one of its core values and just not a smokescreen for what it really values (like maximizing shareholder returns).

Our final point about really valuing ethical governance is of course hard to evidence. But, like trust, confidence in a company's claim to be ethical has to be earned and—as we have seen—can easily be damaged. Ethical governance needs to become part of a company's DNA, not just in product development but across the whole organization from management to marketing. In setting out these pillars of good ethical governance in robotics and AI, we are well aware that implementing these practices will be challenging. As Boddington points out ‘There are indeed very hard questions about how to translate institutional ethical policies into practice’ [61, p. 34]. We are however encouraged to see a very recent example of pressure from within a leading AI company to institute a framework not unlike the one we propose here, as set out in a letter from employees ‘asking leadership to work with employees to implement concrete transparency and oversight process’ [62].

This paper has explored the question of ethical governance in robotics and AI. The paper has argued that ethical governance, while not a singular solution, will be critical to building public trust in robotics and artificial intelligence. It is hard to see how disruptive new IAS technologies such as driverless cars, assistive robots or medical diagnosis AIs will be widely accepted and trusted without transparent, inclusive and agile ethical governance by the organizations that develop and operate them.

## Footnotes

1 Notable because Asimov was undoubtedly the first to establish the principle that robots (and by extension AIs) should be governed by formally stated principles.

2 Including care or workplace assistant robots. For a robot taxonomy, refer to [38, pp. 37–41].

3 Perhaps augmented by a cloud-based AI.

4 Which may be very large, so-called ‘big data’ sets—which themselves pose another problem for verification.

5 Note that the use of the colloquialism ‘black box’ in aviation should not be confused with the ‘black box problem’ in AI, referred to in §3c.

6 Ethical agents should be based on ethical systems other than consequentialism; however, computationally modelling such systems remains a difficult research problem.

## Data accessibility

This article has no additional data.
